# Hospitalization and readmission after single-level fall: a population-based sample

**DOI:** 10.1186/s40621-023-00463-4

**Published:** 2023-10-19

**Authors:** Alan Cook, Rebecca Swindall, Katherine Spencer, Carly Wadle, S. Andrew Cage, Musharaf Mohiuddin, Yagnesh Desai, Scott Norwood

**Affiliations:** 1 Trauma Services, UT Health East Texas, 1020 E. Idel St., Tyler, TX 75701 USA; 2 Department of Epidemiology and Biostatistics, The University of Texas Health Science Center at Tyler, 11937 US Highway 271, Room H252, Tyler, TX 75708 USA; 3 CHRISTUS Health-Texas A&M Spohn Emergency Medicine Residency, Texas A&M University-Corpus Christi, 600 Elizabeth Street, 9B, Corpus Christi, TX 78404 USA; 4 Department of Sports Medicine, The University of Texas at Tyler, 3900 University Blvd., Tyler, TX 75799 USA; 5 Department of Emergency Medicine, UT Health East Texas, 1000 S. Beckham Ave., Tyler, TX 75701 USA

**Keywords:** Geriatric trauma, Falls, Readmission, Hospitalization, Palliative care, Hospital charges

## Abstract

**Background:**

Single-level falls (SLFs) in the older US population is a leading cause of hospital admission and rates are increasing. Unscheduled hospital readmission is regarded as a quality-of-care indication and a preventable burden on healthcare systems. We aimed to characterize the predictors of 30-day readmission following admission for SLF injuries among patients 65 years and older.

**Methods:**

We conducted a retrospective cohort study using the Nationwide Readmission Database from 2018 to 2019. Included patients were 65 and older, admitted emergently following a SLF with a primary injury diagnosis. Hierarchical logit regression was used to model factors associated with readmission within 30 days of discharge.

**Results:**

Of 1,338,905 trauma patients, 65 years or older, 61.3% had a single-level fall as the mechanism of injury. Among fallers, the average age was 81.1 years and 68.5% were female. SLF patients underwent more major therapeutic procedures (56.3% vs. 48.2%), spent over 2 million days in the hospital and incurred total charges of over $28 billion annually. Over 11% of SLF patients were readmitted within 30 days of discharge. Increasing income had a modest effect, where the highest zip code quartile was 9% less likely to be readmitted. Decreasing population density had a protective effect of readmission of 16%, comparing Non-Urban to Large Metropolitan. Transfer to short-term hospital, brain and vascular injuries were independent predictors of 30-day readmission in multivariable analysis (OR 2.50, 1.31, and 1.42, respectively). Palliative care consultation was protective (OR 0.41). The subsequent hospitalizations among those 30-day readmissions were primarily emergent (92.9%), consumed 260,876 hospital days and a total of $2.75 billion annually.

**Conclusions:**

SLFs exact costs to patients, health systems, and society. Transfer to short-term hospitals at discharge, along with brain and vascular injuries were strong predictors of 30-day readmission and warrant mitigation strategy development with consideration of expanded palliative care consultation.

## Introduction

The elderly population, 65 years and older, in the United States (USA), has grown by over a third since 2010; no other age group experienced such a rapid increase (Holleran 2015; https://www.census.gov/newsroom/press-releases/2020/65-olderpopulation-grows.html). There were 54.1 million people aged 65 and older in 2019 in the USA, a 36% increase since 2009 compared to a 3% increase of people under the age of 65 (https://acl.gov/sites/default/files/Aging%20and%20Disability%20in%20America/2020ProfileOlderAmericans.Final_pdf). The elderly population is expected to represent 23.4% of the total population by the year 2060, compared to 15.2% in 2016, people aged 85 and older is expected to triple (https://www.census.gov/library/stories/2018/03/graying-america.html). This leads to an increased population with a higher risk of frailty and disability as the elderly live longer with chronic diseases (Rowe et al. 2016). Further, the elderly have been found to incur higher healthcare costs making up 23% of admissions and 28% of total hospital charges (Richmond et al. 2002).

Falls in the elderly are a major cause of geriatric injury. They are associated with functional decline, and are the leading cause of emergency department (ED) visits, injury, and death (Sterling et al. 2001; Liu et al. 2015; Bonne and Schuerer 2013). From 2011 to 2015, there was a 30% increase in hospital admissions due to falls and a 6% increase in fall patients subsequently admitted to skilled nursing facilities (Khurrum et al. 2021). Falls in the elderly population have the potential for serious injury and poor outcomes leading to potential hospital readmission for subsequent falls (Galet et al. 2018).

As of March of 2023, 65,748,297 people were enrolled in Medicare, an increase of nearly 100,000 in 6 months (https://medicareadvocacy.org/medicare-enrollment-numbers/). Medicare’s annual spending is projected to double from 2019 to 2029, from $782 billion to $1.5 trillion (Chernew et al. 2021). When Medicare was launched in 1965 regulated payments were received with no limits on volume of services and minimal oversight in the coordination of care (Ayanian 2009). In response to concerns over the sustainability of the Medicare system, approaches to contain costs concurrent with quality improvement have been implemented and evaluated (Ayanian 2009; Gupta et al. 2018; Yeung et al. 2021; Vadnais et al. 2020). The rising US elderly population obtaining Medicare coverage, coupled with increasing costs of medical care, accentuate the acute responsibility healthcare professionals have in advancing coordination of health care directed toward tapering preventable readmissions among the elderly.

All-cause 30-day readmission patterns need to be identified and prevented. This study seeks to characterize the inpatient clinical course of geriatric patients, 65 years and older, injured from a single-level fall (SLF). The aim of this study was to characterize the predictors of readmission within 30 days of discharge following a SLF. We hypothesize comorbidity, injury, and discharge patterns among patients interact with index admissions for SLFs and increase risk of all-cause 30-day readmission.

## Methods

We conducted a retrospective cohort study using data from the Nationwide Readmissions Database (NRD) for the years 2018 and 2019. The NRD is a data product of the Healthcare Cost and Utilization Project (HCUP) from the Agency for Healthcare Research and Quality (AHRQ) which is under the US Department of Health and Human Services. The NRD is constructed as a 100 percent sample of hospital discharge data from the 28 and 30 states contributing data to the State Inpatient Databases, for 2018 and 2019, respectively (https://www.hcupus.ahrq.gov/db/nation/nrd/Introduction_NRD_2019.pdf). The inclusion and exclusion criteria, as applied to 2 years of the NRD, produced a very large sample size. As such, all differences in this analysis were statistically significant at the *P* < 0.05 level, unless otherwise indicated. The NRD contains discharge data beginning in 2010; however, a greater sample size would not augment results, as the current 2-year sample size produced significant findings with minimal effect sizes. In addition, by utilizing the NRD from 2018 to 2019 we provide a current report pertaining to trends and costs involving national estimates pertaining to SLFs and 30-day readmission. The hospitals contributing data include all nonfederal short-term general and specialty hospitals, including academic medical centers, whose facilities and care are available to the public (https://www.hcupus.ahrq.gov/db/nation/nrd/Introduction_NRD_2019.pdf). The NRD links successive hospital discharges using a synthetic, anonymous linkage number to track patients across hospitals within a particular state for the given calendar year. In addition to the advantage of linking subsequent hospitalizations, the NRD provides a weighting variable for each discharge record to allow for US population-level inferences. For clarity, when inferences are extrapolated to the population level in this report, they are referred to as “weighted discharges.”

**Fig. 1:**
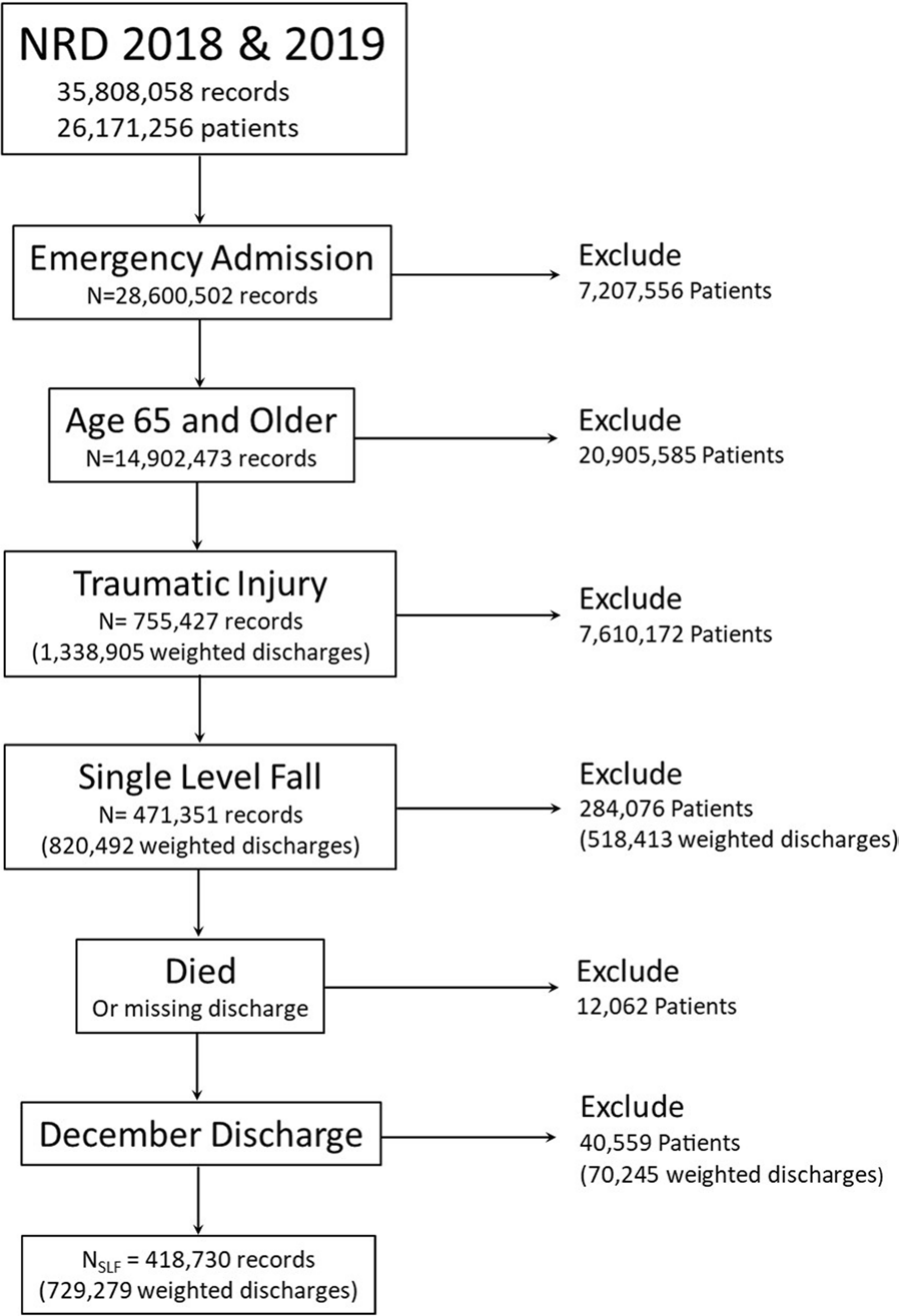
Inclusion and exclusion criteria flow diagram

Inclusion in the study required the patients be 65 years-old or older and their admission classified as non-elective. Patients were classified as having suffered a SLF if their discharge diagnoses included any of the following International Classification of Diseases, Tenth Revision, Clinical Modification (ICD-10-CM) codes: V00381A, V00811A, V00831A, W000XXA, W001XXA, W01XXXA, W03XXXA, W050XXA, W052XXA, W06XXXA, W07XXXA, W08XXXA, W101XXA, W1811XA, W1812XA, W182XXA, W1830XA, W1831XA, W1839XA. Further, patients were considered trauma admissions if their first diagnosis code began with “S” or were T8404*, T794*, T796*, T797*, T79A*, and ended with the characters “A,” “B,” or “C” indicating the initial healthcare encounter for those diagnoses. See Table 1 for definitions of the ICD-10-CM codes. If patients had multiple hospitalizations in the year and none of the hospitalizations were for a SLF, the first hospitalization was selected for comparison. If patients had multiple hospitalizations and one or more were for a SLF, the first SLF hospitalization was included for comparison. Then, to develop estimates of risk factors for readmission, discharge records were excluded if the patient died during the hospitalization. Patients hospitalized in the twelfth month of either 2018 or 2019 were excluded from analysis because the NRD methodology constructs files using one calendar year. Readmissions occurring in subsequent calendar years are not linked to hospitalizations in the prior year(s) (https://www.hcupus.ahrq.gov/db/nation/nrd/Introduction_NRD_2019.pdf). The inclusions and exclusions are enumerated in Fig. 1.

**Table 1:** ICD-10-CM code classification of hospital admission due to a single-level fall

*ICD-10-CM codes*	
V00381A	Fall from other flat-bottomed pedestrian conveyance, initial encounter
V00811A	Fall from moving wheelchair (powered), initial encounter
V00831A	Fall from motorized mobility scooter, initial encounter
W000XXA	Fall on same level due to ice and snow, initial encounter
W001XXA	Fall from stairs and steps due to ice and snow, initial encounter
W03XXXA	Other fall on same level due to collision with another person, initial encounter
W050XXA	Fall from non-moving wheelchair, initial encounter
W052XXA	Fall from non-moving motorized mobility scooter, initial encounter
W06XXXA	Fall from bed, initial encounter
W07XXXA	Fall from chair, initial encounter
W08XXXA	Fall from other furniture, initial encounter
W101XXA	Fall (on)(from) sidewalk curb, initial encounter
W1811XA	Fall from or off toilet without subsequent striking against object, initial encounter
W1812XA	Fall from or off toilet with subsequent striking against object, initial encounter
W182XXA	Fall in (into) shower or empty bathtub, initial encounter
W1830XA	Fall on same level, unspecified, initial encounter
W1831XA	Fall on same level due to stepping on an object, initial encounter
W1839XA	Other fall on same level, initial encounter
*Further trauma admission code if code ends with characters A, B, C (Initial encounter)*	
S	Injuries and external causes
T8404*	Mechanical complication of internal joint prosthesis
T794*	Traumatic shock
T796*	Traumatic ischemia of muscle
T797*	Traumatic subcutaneous emphysema
T79A*	Traumatic compartment syndrome

Elixhauser Comorbidity Readmission Index presented a set of comorbidity measures to predict effects on patient outcomes (Elixhauser et al. 1998), that was further validated (Epstein and Dexter 2017; Mehta et al. 2022), leading to subsequent model development assessing risk of in-hospital mortality and readmission (Moore et al. 2017) provided through the AHRQ HCUP (www.hcup-us.ahrq.gov/toolssoftware/comorbidityicd10/comorbidity_icd10.jsp). The chronic comorbid disease burden was characterized using the Elixhauser Comorbidity Readmission Index by mapping diagnoses ICD-10-CM codes to Elixhauser comorbidity measures table available from the HCUP Elixhauser Comorbidity Software refined for ICD-10-CM diagnoses, v2022.1 (Moore et al. 2017; www.hcup-us.ahrq.gov/toolssoftware/comorbidityicd10/comorbidity_icd10.jsp). The severity of anatomic injury was enumerated as the probability of death estimated by the trauma mortality prediction model (TMPM) for the ICD-10-CM lexicon (Osler et al. 2019). Patients were identified as having had a palliative care consult during their hospitalization if their records included the ICD-10-CM code, Z515, encounter for palliative care. Injuries were similarly identified according to the ICD-10-CM diagnostic code lexicon. Procedures were classified as major diagnostic according to the HCUP Procedure Classes redefined for ICD-10-PCS. Procedures are classified as major based on whether or not they are expected to be performed in an operating room (www.hcup-us.ahrq.gov/toolssoftware/procedureicd10/procedure_icd10.jsp). We also evaluated the characteristics of the subsequent hospitalization among those emergently readmitted within 30 days of discharge from their index SLF hospitalization.

Tests of significance for dichotomous and categorical variables for dichotomous outcomes used the Chi-squared test. Following assessment for normality with the Shapiro–Wilk test (Shapiro and Wilk 1965), normally distributed continuous variables were tested using the t-test for unequal variance. Continuous variables with nonparametric distributions were compared by dichotomous outcomes using the rank sum test. Crude odds ratios were computed to estimate the size and direction of associations of descriptive variables with the outcomes of interest. Hierarchical logit regression was used to model factors associated with 30 days of discharge. The independent variables were selected for inclusion by the process of purposeful selection, described by Hosmer, Lemeshow, and Sturdivant (Hosmer et al. 2013). The treating hospital identifier was included as the random parameter in the models. The most parsimonious models are presented. The model’s ability to discriminate those readmitted within 30 days of discharge from non-readmitted patients was estimated using the area under the receiver operating characteristic curve (Swets et al. 1961). In response to the substantial sample size, resulting in significance associated with limited practical application, variables of interest had effect sizes approaching 30% or incident rates greater than 10%. Statistical calculations were performed using STATA v.17.0 (Stata, Inc., College Station, TX).

## Results

After exclusion of all patients younger than 65 years-old and those whose admission was deemed elective, the remaining 14,902,473 weighted discharges were retained in the group. Traumatic injury accounted for 1,223,877 weighted discharges among elders in the USA for 2018 and 2019. Patients whose emergent admission was due to injuries from a SLF (*n* = 748,544 weighted discharges [61.2%]) were compared to those admitted for other mechanisms of injury (*n* = 475,333 [38.8%]). SLF patients were, on average, older (mean 81.1 years [95% CI 81.0–81.1]) than the non-fall patients (78.6 [95% CI 78.5–78.7]), and had a greater proportion of females, 68.6% (95% CI 68.3–68.9%) versus 58% (95% CI 57.4–58.5%). The majority of the cohort were insured by Medicare/Medicaid (94.1% of SLF and 84.9% of non-SLF). Chronic comorbid conditions were more prevalent in the SLF group. The median (IQR) Elixhauser Comorbidity Readmission Index was 12 (22) among SLF group and 8 (21) among the non-SLF group. The majority of the SLF group were discharged to a skilled nursing, intermediate care facility (67.1%), compared to 54.6% of the non-SLF group. Among the SLF group, 2.6% died in the hospital, whereas 3.6% of the non-SLF patients died.

The rates of all-cause 30-day readmission at any time interval in the dataset were 6.8% greater in the SLF group compared to the non-SLF patient group, 26.1% versus 19.3%, respectively. The median interval to readmission was comparable (43 days versus 41 days) and rate of readmission within 30 days was equivocal (40.7% versus 42.2%). The descriptors of patient zip code income quartile, hospital urban/rural designation, and hospital teaching designation were also similar between SLF and non-SLF patient groups.

There were important differences between groups in terms of therapeutic procedures per person during hospitalization. SLF patients underwent more major therapeutic procedures than the non-SLF patients, 56.3% (95% CI 55.7–56.9%) versus 48.2% (95% CI 47.6–48.7%). The length of stay (LOS), palliative care consults, and hospital costs were equivocal between groups. The total hospital cost for the weighted sample of SLF discharges was estimated to be greater than $28.2 billion annually with a total of 4.6 million days spent in the hospital during the study period. See Table 2.

**Table 2:** Characteristics of 1,223,877 weighted injury hospitalizations among US adults 65 years and older by SLF status

*N*_Weighted_ = 1,223,877	Single-level falls	No single-level fall	*P*
Prevalence, *n*	748,544	475,333	
% (95% CI)	61.2 (60.3–62.0)	38.8 (38.0–39.7)	
Age, years, mean (95% CI)	81.1 (81.0–81.1)	78.6 (78.5–78.7)	
Sex, female, %, (95% CI)	68.6 (68.3–68.9)	58.0 (57.4–58.5)	
Payer status %, (95% CI)			
Medicare/medicaid	94.1 (93.8–94.4)	84.9 (84.3–85.6)	
Private insurance	4.1 (3.8–4.4)	11.4 (10.9–12.0)	
Uninsured	0.3 (0.2–0.4)	0.6 (0.6–0.7)	
No charge	0.02 (0.01–0.04)	0.03 (0.02–0.05)	
Other payer	1.5 (1.4–1.6)	3.0 (2.7–3.2)	
Elixhauser Comorbidity Readmission Index,			
Median (IQR)	12 (22)	8 (21)	
Discharge disposition, %, (95% CI)			
Routine	13.1 (12.8–13.5)	23.8 (23.3–24.4)	
Transfer to short-term hospital	0.9 (0.8–1.0)	1.0 (0.9–1.1)	
Skilled nursing, intermediate care	67.1 (66.6–67.6)	54.6 (54.0–55.3)	
Home health care	15.9 (15.6–16.3)	16.4 (16.0–16.8)	
Left against medical advice	0.4 (0.3–0.5)	0.4 (0.4–0.5)	
Died	2.6 (2.5–2.6)	3.6 (3.5–3.8)	
All-cause readmission, % (95% CI)	26.1 (25.8–26.3)	19.3 (19.1–19.6)	
Days to readmission, median (IQR)	43 (88)	41 (91)	
30-day readmission, %, (95% CI)	40.7 (41.4–42.0)	42.2 (41.8–42.7)	
Zip code income quartile, %, (95% CI)			
$1–$37,999	22.9 (21.9–24.0)	24.6 (23.5–25.8)	
$38,000–$47,999	27.5 (26.6–28.3)	27.9 (27.0–28.8)	
$48,000–$63,999	26.3 (25.5–27.1)	26.1 (25.3–26.9)	
$64,000+	23.3 (22.1–24.6)	21.4 (20.2–22.6)	
Year, %, (95% CI)			0.02
2018	47.8 (46.2–51.3)	49.0 (46.0–52.0)	
2019	51.3 (48.6–53.8)	51.0 (48.0–54.0)	
Hospital urban/rural designation, %, (95% CI)			
Large metropolitan, > 1 Million population	52.6 (50.0–55.1)	51.1 (48.1–54.1)	
Small metropolitan, < 1 Million population	39.2 (36.6–41.7)	40.5 (37.5–43.6)	
Micropolitan, 10,000–50,000 population	6.6 (5.9–7.5)	6.5 (5.7–7.4)	
Non-urban	1.6 (1.4–1.9)	1.9 (1.6–2.1)	
Hospital teaching designation, %, (95% CI)			
Metropolitan, non-teaching	21.0 (19.3–22.7)	17.5 (15.9–19.2)	
Metropolitan, teaching	70.7 (68.8–72.6)	74.1 (72.1–76.1)	
Non-metropolitan	8.3 (7.5–9.2)	8.4 (7.5–9.3)	
Major therapeutic procedures, %, (95% CI)	56.3 (55.7–56.9)	48.2 (47.6–48.7)	
Length of stay, days, median (IQR)	4 (3)	4 (4)	
Total hospital days	4,588,088	3,147,963	n/a
Palliative care consult, % (95% CI)	4.2 (4.1–4.3)	4.6 (4.4–4.7)	
Hospital charge, $, median (IQR)	$52,538 (57,584)	$50,492 (62,165)	

*P* value < 0.001 for all comparisons except where noted

**0.01 < *P* value < 0.001

Next, the cohort was restricted to the 729,279 patients aged 65 years and older who were emergently admitted for treatment of traumatic injuries sustained from a SLF and survived their index hospitalization. We compared this group of patients according to their readmission status. From this group, 85,287 (11.7%) were readmitted within 30 days. Patients readmitted were slightly older, on average, 81.3 years versus 81.0 years in the no readmission group. The OR of 1.006 indicates an increase in the odds of readmission of 6% for each 10-year increase when greater than 65 years of age. There were fewer females in the 30-day readmission group relative to the non-readmitted group, 62.1% versus 69.9%, with an unadjusted OR of 0.69 (95% CI 0.68–0.71). Similarly, compared to Medicare/Medicaid insurance, private insurance and uninsured status were negatively associated with 30-day readmission (private insurance OR 0.83 [95% CI 0.79–0.88] uninsured OR 0.75 [95% CI 0.62–0.90]).

When injury categories were compared for association with readmission, a majority of the categories had *P* values ≤ 0.05, effect sizes, as indicated by crude ORs, were modest. Injuries with the largest unadjusted odds of 30-day readmission included the urinary system (kidneys, ureter, bladder, or urethra) (OR 1.38 [95% CI 1.17–1.64]), vascular injury (OR 1.35 [95%CI 1.10–1.65]), traumatic brain injury (TBI) (OR 1.31 [95% CI 1.28–1.34]), intra-abdominal solid organ (pancreas, liver, spleen, or adrenal) (OR 1.25 [95% CI 1.08–1.46]), spinal cord injuries (OR 1.29 [95% CI 1.16–1.44]), and skull fracture (OR 1.22 [95% CI 1.11–1.33]). The collective burden of injury was greater among the 30-day readmission group, with a median (IQR) TMPM probability of death of 2.3% (1.4%) compared to the non-readmitted group, 2.1% (1.4%), unadjusted OR 1.12 (95% CI 1.10–1.13).

Similar to the injury categories, a majority of the comorbid chronic diseases demonstrated associations with 30-day readmission with *P* values ≤ 0.05 and modest effect sizes. Severe liver disease and severe renal failure showed the highest unadjusted odds of 30-day readmission, 2.20 (95% CI 1.99–2.43) and 2.18 (95% CI 2.10–2.25), respectively. A patient with previous heart failure was 1.75 (95% CI 1.72–1.79) times more likely to be readmitted and 1.66 (95% CI 1.62–1.69) times if the patient had complicated hypertension. Comorbid conditions further involving the circulatory system (anemia, coagulopathies, chronic pulmonary disease, valvular disease) increase readmission risk from 30 to 42%. There was a 54% increased risk of readmission among patients with complicated diabetes (95% CI 1.51–1.58). Moderate renal disease also increased risk of readmission by 35% (95% CI 1.32–1.38). Patients with obesity (9.4%) and patient weight loss (9.5%) were 22% and 35% more likely to be readmitted, respectively. When the accumulated burden of comorbidity was compared between groups with the Elixhauser Comorbidity Index for Readmission, the median (IQR) value for the readmitted group was 5 points higher, 16 (25) versus 11 (20), with an unadjusted OR of 1.03 (95% CI 1.03–1.03). Demonstrating a 1-point increase in Elixhauser Comorbidity Index for Readmission score increased the chances for readmission by 3%.

The patients’ discharge dispositions were associated with the likelihood of being readmitted within 30 days of discharge from the acute hospitalization. Compared to routine discharge to home, patients transferred to a short-term hospital were 2.7 times more likely to be readmitted and patients leaving against medical advice were 2.1 times more likely, 95% CI 2.46–2.89 and 1.84–2.34, respectively. SLF patients were more likely to be discharged to a skilled nursing facility (SNF) (72.9%) compared to other discharge destinations, with a 39% increased odds of 30-day readmission compared to routine discharge home (95% CI 1.35–1.43). Increasing home zip code income quartile among fallers was associated with decreasing odds of being readmitted within 30 days, though once again effect size was modest, with the highest income quartile being 9% less likely to be readmitted compared to the lowest quartile. Comparing discharge from hospitals in large, metropolitan areas, successively less dense population concentrations were also associated with decreasing odds of readmission within 30-day. Hospitals in small metropolitan areas, micropolitan areas, and non-urban areas were associated with unadjusted ORs of 0.97, 0.88, and 0.84, respectively. The median (IQR) LOS was longer among the 30-day readmit patient group, 5 (4) versus 4 (3). Palliative care consults were less common in the readmitted patient group, 1.6% versus 3.0% (OR 0.56 [95% CI 0.52–0.61]. See Table 3.

**Table 3:** Characteristics of 729,279 SLF-injured adults, 65 years-old and older by 30-day all-cause readmission status*

	30-day readmission	No readmission	*P*	Crude OR (95% CI)	*P*
Prevalence, *n*	85,287	643,993			
% (95% CI)	11.7 (11.6–11.8)	88.3 (88.2–88.4)			
Emergency readmission, % (95% CI)	92.9 (92.7–93.1)	n/a	–	n/a	–
Age, years, mean (95% CI)	81.3 (81.2–81.4)	81.0 (80.9–81.0)		1.006 (1.004–1.007)	
Gender, female, %, (95% CI)	62.1 (61.6–62.6)	69.9 (69.7–70.2)		0.69 (0.68–0.71)	
Payer status %, (95% CI)					
Medicare/medicaid	95.0 (94.6–95.3)	94.0 (93.7–94.3)		Referent	
Private insurance	3.5 (3.2–3.8)	4.2 (3.9–4.5)		0.83 (0.79–0.88)	
Uninsured	0.2 (0.2–0.3)	0.3 (0.2–0.4)		0.75 (0.62–0.90)	**
No charge	0.02 (0.01–0.05)	0.02 (0.01–0.04)		0.93 (0.53–1.66)	††
Other payer	1.3 (1.2–1.4)	1.5 (1.4–1.6)		0.83 (0.76–0.90)	
Injury categories, % (95% CI)					
Skull fracture, ± brain injury	1.2 (1.1–1.3)	1.0 (0.9–1.0)		1.22 (1.11–1.33)	
Rib and/or sternal fractures	7.4 (7.1–7.7)	7.3 (7.1–7.6)		1.03 (0.99–1.07)	††
Cervical spine fractures	2.8 (2.6–3.1)	2.8 (2.7–3.0)		1.03 (0.98–1.09)	
Thoracic spine fractures	3.6 (3.4–3.8)	3.2 (3.1–3.3)		1.12 (1.07–1.18)	
Lumbar spine fractures	4.2 (4.0–4.4)	3.9 (3.8–4.0)		1.11 (1.06–1.16)	
Pelvis fractures	6.3 (6.0–6.5)	7.2 (7.1–7.3)		0.85 (0.82–0.88)	
Humerus, radius, ulna, wrist, hand fracture	9.6 (9.4–10.0)	10.2 (10.0–10.4)		0.94 (0.91–0.97)	
Femur, patella, tibia, fibula, ankle, foot fracture	52.8 (52.0–53.6)	55.7 (55.0–56.4)		0.88 (0.87–0.90)	
Traumatic brain injuries, ± coma	16.8 (16.2–17.4)	13.4 (13.0–14.0)		1.31 (1.28–1.34)	
Pancreas, liver, spleen, adrenal inj	0.4 (0.3–0.5)	0.3 (0.3–0.4)	**	1.25 (1.08–1.46)	**
Kidneys, ureter, bladder, urethra	0.3 (0.3–0.4)	0.2 (0.2–0.3)		1.38 (1.17–1.64)	†
Open wounds	8.6 (8.3–8.9)	8.0 (7.8–8.2)		1.07 (1.04–1.11)	
Vascular injuries	0.2 (0.2–0.3)	0.2 (0.1–0.2)	**	1.35 (1.10–1.65)	**
Spinal cord injuries	0.8 (0.7–0.9)	0.6 (0.6–0.7)		1.29 (1.16–1.44)	
Superficial contusions/abrasions	14.4 (14.0–14.9)	13.6 (13.3–13.9)		1.07 (1.04–1.10)	
Comorbid conditions, % (95% CI)					
Autoimmune disorders	4.8 (4.6–5.0)	4.5 (4.4–4.6)		1.07 (1.02–1.12)	
Anemia deficiency	25.4 (24.8–25.9)	19.9 (19.6–20.3)		1.37 (1.34–1.40)	
Coagulopathies	10.1 (9.8–10.5)	7.4 (7.2–7.5)		1.42 (1.38–1.47)	
Dementia	25.3 (24.8–25.8)	24.0 (23.8–24.2)		1.07 (1.05–1.10)	
Depression	16.1 (15.6–16.5)	15.9 (15.6–16.1)		1.01 (0.99–1.04)	
Diabetes, chronic complications	21.1 (20.6–21.5)	14.9 (14.7–15.1)		1.54 (1.51–1.58)	
Diabetes, without complications	11.7 (11.4–12.1)	11.9 (11.7–12.1)	††	0.99 (0.96–1.02)	††
Heart failure	26.4 (25.9–26.9)	17.0 (16.8–17.3)		1.75 (1.72–1.79)	
Hypothyroidism	23.5 (23.0–26.9)	23.1 (22.9–23.4)		1.01 (0.99–1.79)	
Hypertension, complicated	40.0 (39.3–40.6)	28.7 (28.3–29.0)		1.66 (1.62–1.69)	
Hypertension, uncomplicated	44.2 (43.6–44.8)	51.0 (50.6–51.3)		0.76 (0.75–0.78)	
Chronic pulmonary disease	25.7 (25.3–26.2)	20.5 (20.2–20.7)		1.35 (1.32–1.38)	
Neurological disorders affecting movement	6.1 (5.9–6.3)	5.8 (5.7–5.9)		1.08 (1.03–1.12)	
Obesity	9.4 (9.1–9.8)	8.0 (7.8–8.2)		1.22 (1.18–1.26)	
Neurological disorders, other	11.8 (11.4–12.1)	8.9 (8.7–9.1)		1.37 (1.33–1.41)	
Paralysis	5.0 (4.8–5.2)	4.2 (4.1–4.3)		1.19 (1.14–1.24)	
Peripheral vascular disease	9.0 (8.7–9.4)	7.5 (7.2–7.7)		1.24 (1.20–1.28)	
Renal failure, moderate	19.9 (19.5–20.4)	15.6 (15.3–15.8)		1.35 (1.32–1.38)	
Renal failure, severe	8.6 (8.3–8.9)	4.2 (4.1–4.3)		2.18 (2.10–2.25)	
Valvular heart disease	12.7 (12.3–13.0)	10.1 (9.9–10.3)		1.30 (1.27–1.34)	
Weight loss	9.5 (9.1–9.9)	7.3 (7.0–7.5)		1.35 (1.31–1.40)	
Liver disease, severe	1.0 (0.9–1.1)	0.4 (0.4–0.5)		2.20 (1.99–2.43)	
TMPM probability of death, %, median (IQR)	2.3 (1.4)	2.1 (1.4)		1.12 (1.10–1.13)	
Elixhauser comorbidity index, readmission					
Median (IQR)	16 (25)	11 (20)		1.03 (1.03–1.03)	
Discharge disposition, %, (95% CI)					
Routine	10.7 (10.2–11.0)	13.8 (13.5–14.2)		Referent	
Transfer to short-term hospital	1.6 (1.5–1.8)	0.8 (0.8–0.9)		2.66 (2.46–2.89)	
Skilled nursing, intermediate care	72.9 (72.3–73.5)	68.3 (67.8–68.8)		1.39 (1.35–1.43)	
Home health care	14.2 (13.7–14.6)	16.6 (16.3–17.0)		1.12 (1.08–1.16)	
Left against medical advice	0.6 (0.5–0.8)	0.4 (0.3–0.5)		2.08 (1.84–2.34)	
Year, %, (95% CI)					
2018	49.0 (46.3–51.7)	48.7 (46.1–51.3)			
2019	51.0 (48.3–53.7)	51.3 (48.7–53.8)			
Zip code income quartile, %, (95% ci)					
$1–$37,999	24.0 (22.9–25.2)	22.8 (21.8–23.8)		Referent	
$38,000–$47,999	27.4 (26.4–28.3)	27.5 (26.6–28.3)		0.94 (0.92–0.97)	
$48,000–$63,999	25.8 (24.9–26.7)	26.3 (25.6–27.2)		0.92 (0.90–0.95)	
$64,000+	22.8 (21.5–24.1)	23.4 (22.2–24.7)		0.91 (0.89–0.94)	
Hospital urban/rural designation, %, (95% CI)					
Large metropolitan, > 1 million population	53.8 (51.1–56.4)	52.5 (49.9–55.0)		Referent	
Small metropolitan, < 1 million population	38.6 (36.0–41.2)	39.1 (36.6–41.7)		0.97 (0.95–0.99)	
Micropolitan, 10,000–50,000 population	6.1 (5.3–7.1)	6.7 (5.9–7.6)		0.88 (0.84–0.92)	
Non-urban	1.5 (1.2–1.7)	1.7 (1.4–1.9)		0.84 (0.76–0.92)	
Hospital teaching designation, %, (95% CI)					
Metropolitan, non-teaching	20.9 (19.2–22.8)	21.1 (19.5–22.9)		Referent	
Metropolitan, teaching	71.4 (69.4–73.4)	70.5 (68.5–72.4)		1.01 (0.99–1.04)	††
Non-metropolitan	7.6 (6.8–8.5)	8.4 (7.6–9.3)		0.89 (0.86–0.93)	
Major therapeutic procedures, %, (95% CI)	54.4 (53.7–55.2)	56.9 (56.3–57.5)		0.90 (0.88–0.92)	
Length of stay, days, median (IQR)	5 (4)	4 (3)		1.02 (1.02–1.02)	
Palliative care consult, %, (95% CI)	1.6 (1.5–1.8)	3.0 (2.9–3.1)		0.56 (0.52–0.61)	
Hospital charge, $, median (IQR)	59,684 (68,626)	53,362 (58,685)		1.00 (1.00–1.00)	

*P* value < 0.001 for all comparisons except where noted

**0.01 < *P* value < 0.001

^†^0.05 < *P* value < 0.01

^††^*P* > 0.05

The hierarchical logit regression model demonstrated varying associations with 30-day readmission among predictors related to patient demographics, injury categories, procedure classes, and discharge disposition. Similar to univariate analysis age also showed a positive association with 30-day readmission with an OR of 1.006 (95% CI 1.005–1.009). The indices of injury severity (TMPM probability of death) and the Elixhauser Comorbidity Index for Readmission were each positively associated with increasing odds of 30-day readmission, OR 1.03 (95% CI 1.01–1.05) and 1.03 (95% CI 1.03–1.03), respectively. Interestingly, the variable female sex demonstrated a phenomenon known as Simpson’s paradox (Simpson 1951) whereby the univariate analyses demonstrated negative associations with 30-day readmission, then with the presence of confounders in the multivariable regression model, demonstrate positive associations, OR 1.29, [95% CI 1.26–1.32]. Palliative care consultation continued to demonstrate a protective effect, OR 0.41 (95% CI 0.37–0.44).

Among injury categories, the strongest predictors of 30-day readmission were the presence of vascular injury, brain injury, and injury to the urinary system (OR [95% CI] 1.42 [1.13–1.78], 1.31 [1.27–1.35], 1.25 [1.04–1.50], respectively). Among the comorbid conditions, obesity was associated with the largest effect size, OR 1.22 (95% CI 1.18–1.27). Conversely, the category, neurological disorders, other, was negatively associated (OR 0.15, [95% CI 0.14–0.16]). Transfer to a short-term hospital and leaving against medical advice upon discharge for injuries from SLF presented the strongest predictor of 30-day readmission (OR 95% CI 2.50 [2.25–2.78] and 2.18 [1.86–2.55]). The regression model demonstrated a modest degree of discrimination, area under the receiver operating characteristic curve of 0.65. See Table 4.

**Table 4:** Hierarchical logit model for predictors of readmission within 30 days among trauma patients 65 and older admitted for single-level fall

	Odds ratio	95% CI	*P*
Trauma mortality prediction model^§^	1.03	1.01–1.05	
Elixhauser comorbidity index for readmission	1.03	1.03–1.03	
Female	1.29	1.26–1.32	
Age, years	1.007	1.005–1.009	
Palliative care consult	0.41	0.37–0.44	
Injury categories			
Pelvis fracture	0.90	0.86–0.94	
Sprain or strain	0.87	0.80–0.94	
Upper extremity fracture	1.04	1.00–1.08	
Traumatic brain injury, ± coma	1.31	1.27–1.35	
Kidneys, ureter, bladder, urethra	1.25	1.04–1.50	†
Eyes, ears, nasopharynx, oropharynx, teeth	0.87	0.81–0.93	
Vascular injuries	1.42	1.13–1.78	†
Comorbid conditions			
Alcohol abuse	0.88	0.83–0.93	
Deficiency anemia	0.88	0.86–0.91	
Cancer, lymphoma	0.81	0.73–0.90	
Cancer, metastatic	0.71	0.65–0.77	
Cerebrovascular disease	0.91	0.86–0.95	
Dementia	1.05	1.03–1.09	
Depression	0.92	0.89–0.95	
Diabetes mellitus without complications	0.94	0.91–0.97	
Drug abuse	0.85	0.78–0.93	
Neurological disorders affecting movement	1.05	1.01–1.10	†
Neurological disorders, other	0.15	0.14–0.16	
Seizures and epilepsy	1.11	1.05–1.16	
Obesity	1.22	1.18–1.27	
Psychoses	0.89	0.84–0.94	
Major therapeutic procedure	0.97	0.96–0.99	
Minor therapeutic procedure	1.04	1.03–1.05	
Discharge disposition			
Routine	Referent	Referent	
Transfer to short-term hospital	2.50	2.25–2.78	
Skilled nursing, intermediate care	1.35	1.30–1.40	
Home health care	1.06	1.01–1.10	
Left against medical advice	2.18	1.86–2.55	

*P value < 0.001 for all comparisons except where noted

**0.01 < *P* value < 0.001

^†^0.05 < *P* value < 0.01

§Logit transformation of trauma mortality prediction model probability of death (pDeath) = $\log ( \, p{\text{Death /}}(1 - p{\text{Death}}))$

Of the patients readmitted within 30 days of their index hospitalization for a SLF, 92.3% (*n* = 82,443 weighted discharges) were categorized as emergent. Of these, 12.2% included new diagnoses of traumatic injury. A lower proportion were discharged to short-term hospitals and a higher proportion to home with home health services. Of note, 7% died during the following hospitalization (95% CI 6.8–7.3%). In the subsequent hospitalization, 31% underwent major therapeutic procedures. The median hospital length of stay was 5 days (IQR 5), with median charges of $41,606 (IQR $54,742). The subsequent hospitalization consumed a total of 521,752 hospital days and a total of $5.5 billion. See Table 5.

**Table 5:** Characteristics of emergent readmission hospitalization

Prevalence, *n*, (%)*	82,433 (92.9)
New diagnosis traumatic injury, %, (95% CI)	12.2 (11.8–12.6)
Payer status %, (95% CI)	
Medicare/medicaid	94.7 (94.3–95.0)
Private insurance	3.6 (3.3–3.9)
Uninsured	0.2 (0.2–0.3)
No charge	0.02 (0.01–0.04)
Other payer	1.5 (1.3–1.7)
Discharge disposition, %, (95% CI)	
Routine	10.0 (9.7–10.4)
Transfer to short-term hospital	1.1 (1.0–1.3)
Skilled nursing, intermediate care	62.1 (61.5–62.6)
Home health care	18.7 (18.3–19.24)
Left against medical advice	0.4 (0.3–0.5)
Died	7.0 (6.8–7.3)
Major therapeutic procedures, %, (95% CI)	31.0 (30.3–31.7)
Length of stay, days, median (IQR)	5 (5)
Total hospital days	521,752
Palliative care consult, % (95% CI)	11.8 (11.4–12.1)
Hospital charge, $, median (IQR)	$41,606 (54,742)
Hospital charge, $, sum	$5,453,048,293

*Percentage of all patients (*n* = 88,738) readmitted within 30 days of discharge from index hospitalization for SLF

## Discussion

Single-level falls represent the majority of hospitalizations for traumatic injury in the USA among patients 65 years of age and older (Holleran 2015; Bonne and Schuerer 2013; DiMaggio et al. 2016; Fisher et al. 2017). Moreover, these same patients consume a substantial share of the US healthcare cost (Richmond et al. 2002). With falls being a top cause of elderly trauma, and rates of falls increasing with age (Khurrum et al. 2021), as the population of the elderly increases (https://www.census.gov/newsroom/press-releases/2020/65-older-population-grows.html; https://acl.gov/sites/default/files/Aging%20and%20Disability%20in%20America/2020ProfileOlderAmericans.Final_pdf; https://www.census.gov/library/stories/2018/03/graying-america.html) continued efforts to define clinical characteristics of SLFs among the elderly may prove beneficial. By analyzing patients of the NRD, we were able to define characteristics pertaining to age, sex, payer status, injury patterns, comorbidities, discharge disposition and cost associated with SLFs, and risk of 30-day readmission.

The SLF patient groups were on average older and there were more women than men. Analysis of 30-day readmissions among SLF patients produced contradictory results pertaining to sex. Similar studies have reported conflicting results. A cost exploration of readmissions found females were 87% less likely to be readmitted (Carey and Stefos 2016). An assessment of mortality among SLF patients, reported females were more likely to be readmitted following a SLF, of note, the likelihood of males being readmitted trended upward over the study period (Galet et al. 2018). Jenks et al. (2009) explored hospital readmissions among Medicare recipients and found no real distinction between sex and readmission.

Furthermore, no meaningful differences were found between traumatic injury and zip code income quartiles, indicating risk of trauma crosses socioeconomic lines. Howbeit, as income increased the probability of readmission decreased, jointly, less dense populations predicated a protective effect. Begging the question, is it rurality or poverty that determines outcomes (Weber et al. 2005)? Age, when a patient is older than 65, no matter socioeconomic status or residency density, experience a steady increase in risk of SLF, coupled with increased frailty and comorbidities, exacerbate the risk of readmission (Holleran 2015; Khurrum et al. 2021; Goodmanson et al. 2012). Upon discharge, a patients fragility and assessment of comorbid conditions are typically part of the of the care coordination process (Hatcher et al. 2019; Rubenstein 2006). Equitable health care is a crucial second component (Chokshi 2018), consideration of a patient’s home life and ability to follow discharge instruction is paramount.

More than half of the SLF patient population required major therapeutic procedures. Major therapeutic procedures, according to the HCUP procedure classes include, but are not limited to, procedures involving the brain, spine/back, open-approach repairs of upper or lower extremities, and arteries, veins, nerves or muscles of the face, neck, arms, back, and legs. Trauma to the head, face, and neck is severe, costly and commonly reported as an associated injury following a SLF among the elderly (Bergeron et al. 2006; Rau et al. 2014). Severe extremity fractures are likewise a frequent and costly injury reported among elderly SLFs (Khurrum et al. 2021; Gelbard et al. 2014; Newgard et al. 2005). Considering the aforementioned procedure classes, and prevalence among fallers, it is of interest that injuries of readmitted patients with effect sizes large enough to warrant closer consideration also involved: the vascular system and TBI. A similar study of the NRD exploring falls and readmissions reported falls and readmission rates had increased over time but did not report on injury (Galet et al. 2018). Identification of high risk injury patterns following a fall and subsequent 30-day readmission informs clinical practice and expedites increased levels of coordination among at risk patients.

SLF patients had higher rates of comorbidities and were more likely to be readmitted. Galet et al. (2018), also using the NRD database from 2010 to 2014, reported similar associations between comorbidities and admission for a fall injury. Comorbid conditions are a source of morbidity and mortality among the elderly and affect how well a patient can recover from a traumatic injury (Holleran 2015). Recovery from traumatic injury in the context of comorbid conditions is beset with potential complications and becomes a major factor when determining treatment following trauma among elderly patients (Richmond et al. 2002; Bonne and Schuerer 2013). Comorbid conditions involving the cardiovascular and renal systems, diabetes and obesity are common and require increased levels of patient care coordination. For example, a matched case control study found obese patients were 1.25 times more likely to be readmitted, yet the reasons for readmission were similar to the nonobese patients, indicating a broad range of interventions following discharge may be warranted for obese patients (Reinke et al. 2012).

Following a SLF, a majority of patients were discharged to a SNF. An exploration of the National Trauma Data Bank from 2011 through 2015 found patients discharged to a SNF following a SLF increased from 51 to 57% over the study period (Khurrum et al. 2021). Richmond et al. (2002), reported with increasing age, total number of injuries, and injuries due to a fall, patients were more likely to be discharged to a SNF. Prabhakaran et al. (2020), also exploring the NRD for predictors of readmission following a fall in 2020, similarly reported increased risk of readmission when discharged to a SNF or short-term hospital.

In response to SNF discharges increasing over time and discharge to SNFs association with increased risk of 30-day hospital readmission, Mileski et al. (2017) targeted quality improvement initiatives aimed to decrease these rates of readmission. Themes associated with reduction of 30-day readmission included specialized staff (13%), quality improvement models (11%), and collaborative case management (10%) (Mileski et al. 2017). The top two barriers were implementation (17%) and quality improvement tracking (17%) (Mileski et al. 2017). Burke et al. (2017), performed a qualitative analytical approach, informed by Social Constructivist Theory, across three hospitals, utilizing purposeful selection, 25 clinicians were interviewed. Central themes tied to decision making involving evaluation and discharge decision making were found where clinicians described pressure to be expeditious resulting in underinformed and overuse of SNFs as a “safety net” for older adults (Burke et al. 2017).

Of note, the retrospective nature of the data assumes all discharges have an equal chance for readmission, which limits interpretation, as mortality following discharge may over stimulate findings. Emergent readmissions following a SLF for a new diagnosis of trauma were not uncommon. Utilizing data from the HCUP, Friedman et al. ( 2004), analyzed discharges among residents across four States. They found approximately 19% of patients with a preventable index admission had at least one preventable readmission within 6 months, with some evidence suggesting preventable readmissions may partially reflect the complexity of underlying problems (Friedman and Basu 2004). An exploration of older adults patterns of use, adverse outcomes and effectiveness of interventions in the ED, found older Americans have distinct patterns of service use and care needs and current disease-oriented and episodic models of emergency care do not meet older patient’s needs (Aminzadeh and Dalziel 2002). Older patients who fell are at increased risk of preventable readmission, clinicians must consider the entire patient/injury/comorbidity profile to inform care coordination.

SLFs among the elderly and preventable all-cause 30-day readmissions are common and costly. In 2015, data from the Medicare Current Beneficiaries Survey for non-fatal falls was analyzed and it was estimated the annual hospital, physician and other health professionals costs, following a fall was $23 billion, with Medicare and Medicaid paying 75.2% (Florence et al. 2018). The median post fall and baseline expenditures of a single SLF among 51 EDs in 7 northwest counties in 2011 were explored to examine increases in charges following a fall (Newgard et al. 2021). Post fall annual medical costs were found to be $26,143, an increase from the previous year baseline costs of $8,642, with higher costs being associated with older age, comorbidities, extremity fractures, non-injury diagnoses, and surgical interventions (Newgard et al. 2021). In 2004, Friedman et al. (2004) explored rates and cost of 17 prevention quality indicator conditions across 4 states and found hospital cost from preventable readmission to be $7.3 million over a 6-months period. Then, Friedman et al. (2008) explored all hospital discharge charges of adults across six states in 2008, and reported that readmissions more than double the cost of healthcare. We provide a successive nationwide estimate pertaining to the financial burden of SLFs and readmission after a fall.

Attempts to restrain hospital readmission among Medicare recipients exist within the Affordable Care Act, where the Hospital Readmission Reduction Program (HRRP) was enacted in 2012. The HRRP is tasked to encourage hospitals to improve coordination of care and better engage patients and caregivers; however, initial reductions in readmissions plateaued upon implementation (Desai et al. 2016). During the first 3 years of the implementation of the HRRP major teaching hospitals, hospitals with lower income and Medicare beneficiaries were more likely to incur penalties due to higher rates of readmission (Boccuti and Casillas 2015). A statistical brief from the AHRQ, reporting on the characteristics of all-cause 30-day readmission from the NRD from 2010 to 2016, found that while Medicare patients readmission rates decreased by 7% during the study period, readmission was still twice as high as their counterparts (Bailey et al. 2006). Over a decade after the HRRP implementation, we found that not only were most of the patients Medicare beneficiaries they were still more likely to be readmitted.

Healthcare utilization reduction through fall prevention among the elderly has long been studied. Risk factors identified include: sedative use, cognitive impairment, disability of the lower extremities, abnormalities of balance/gait and foot problems, with the risk of falling increasing linearly with the number of risk factors (Tinetti et al. 1988; Carpenter et al. 2009). Up to 71% of trauma care programs involve combinations of fall risk screening assessment and physical therapy/exercise; however, the reviewed screening tools were found to be inaccurate or not feasible for usage in the ED (Hammouda et al. 2021). Prevention programs have been proven to be effective; however, evidence of the effectiveness of interventions is limited (Bleijlevens et al. 2010). Campaigns have been created like Preventing Falls: A Guide to Implementing Effective Community-Based Fall Prevention Programs created by the CDC in 2015 (Frieden et al. 2015). As well the emerging global care model, community paramedicine, with a framework built around prevention efforts and chronic care management (Quatman-Yates et al. 2022). Moving forward, prospective studies implementing and measuring the effect of these established program are called for.

When traumatic injury is not prevented, ensuring smooth care transition upon discharge can prevent health status declines that often lead to readmission (Silow-Carroll et al. 2011). Fisher et al. (2017) discussed how ongoing care and rehabilitation needs of older patients may be best met through comprehensive geriatric assessment that may facilitate informed early decision making. Gaps in discharge planning, lack of communication, and delays in post care are the specific shortcomings in healthcare that have been found to contribute to readmissions (Bisognano and Boutwell 2009). Hospitals that clarify discharge instructions coordinate with post-acute care providers and primary care physicians reduced medical complications and as a result were found to reduce hospital readmissions (Boccuti and Casillas 2015). Careful inclusion of caregivers in these coordination of care instructions has been explored and patients involved in relational coordination between providers and caregivers were positively associated with improved outcomes (Friedman et al. 2008; Weinberg et al. 2007).

A well-known coordination of care program involves palliative care, created to provide specialized medical care for people living with chronic and serious illnesses or diseases. Since its introduction in the 1980’s (Carlson et al. 1988), there has been a linear increase in the number of palliative care programs in hospital systems (Campbell 2006). The literature shows hospital palliative care programs demonstrate improved patient and system outcomes (Campbell 2006; Schenker and Arnold 2015). We found that increasing palliative care consultations may improve outcomes following a SLF and potentially reduce 30-day readmissions. Research involving palliative care following traumatic injury and reduction of subsequent readmission has been explored in a variety of scenarios including, but not limited to: comparisons of the seriously injured (Enguidanos et al. 2012) and propensity score matching (O'Connor et al. 2015; Ranganathan et al. 2013), each reporting that palliative care effectively reduced rates of 30-day readmissions. How these palliative care consultations impact outcomes were explored: goal care discussions was associated with lower readmission rate, however, symptom management was not (O'Connor et al. 2015). Greatest effects of readmission prevention were found if palliative care was received within 6 days of index admission (Barkley et al. 2019).

However, with increasing usage of services, the current palliative care models need to evolve in order to remain an effective intervention during times of serious illness and transitionary care (Schenker and Arnold 2015). The potential for a reduction in 30-day hospital readmission in the presence of palliative care and feasibility of increased usage of services warrants further investigation. Preventions of SLFs are of paramount importance, followed by the need for effective coordination of care designed to meet the individual needs of each patient to ensure they are not subsequently readmitted.

## Limitations

The results and conclusions of this study must be taken in light of certain limitations. First, our results are drawn from hospital discharge records rather than data gathered for the question under study. Next, the NRD does not abstract deaths occurring outside of hospitals. As such, the rates of readmission we report may be an overestimation because the denominator assumes all who are discharged alive are equally at risk for readmission. Finally, by virtue of the large sample size, the p values we report are small even for trivial effect sizes. Therefore, we have focused the reported results on those with effect sizes of clinical or administrative significance.

## Conclusion

Single-level falls exact a substantial toll on wellbeing of elderly persons, as well as exerting a sizeable burden on the healthcare system and society at large. There are several opportunities to mitigate the added burden of readmission following hospitalization for SLFs. These include identifying vulnerable groups of patients for whom optimizing discharge disposition and aftercare coordination, along with clarification of goals of care and patient/family expectations may decrease the risk of readmission.

## Data Availability

The dataset used and analyzed in the current study, the Nationwide Readmission Database for 2018 and 2019, is publicly available from the Healthcare Cost and Utilization Project at https://hcup-us.ahrq.gov/nrdoverview.jsp.

## Abbreviations

US: United States
ED: Emergency department
HRRP: Hospital Readmission Reduction Program
SLF: Single-level fall
NRD: National Readmissions Database
HCUP: Healthcare Cost and Utilization Project
AHRQ: Agency for Healthcare Research and Quality
ICD-10-CM: International Classification of Diseases, Tenth Revision, Clinical Modification
TMPM: Trauma mortality prediction model
LOS: Length of stay
TBI: Traumatic brain injury
SNF: Skilled nursing facility
